# Low-intensity extracorporeal shockwave therapy in the treatment of erectile dysfunction – a narrative review

**DOI:** 10.1590/S1677-5538.IBJU.2023.9904

**Published:** 2023-05-15

**Authors:** Rodrigo R. Vieiralves, Mathias Ferreira Schuh, Luciano Alves Favorito

**Affiliations:** 1 Universidade do Estado do Rio de Janeiro - UERJ Unidade de Pesquisa Urogenital Rio de Janeiro RJ Brasil Unidade de Pesquisa Urogenital - Universidade do Estado do Rio de Janeiro - UERJ, Rio de Janeiro, RJ, Brasil

**Keywords:** Erectile Dysfunction, Penis, Extracorporeal Shockwave Therapy, Randomized Controlled Trials as Topic

## Abstract

**Objectives:**

To provide an overview of low-intensity extracorporeal shockwave therapy (LIEST) for erectile dysfunction (ED), pointing out which concepts are already consolidated and which paths we still need to advance.

**Materials and Methods:**

We performed a narrative review of the literature on the role of shockwave therapies in erectile dysfunction, selecting publications in PUBMED, including only relevant clinical trials, systematic reviews and meta-analyses.

**Results:**

We found 11 studies (7 clinical trials, 3 systematic review and 1 meta-analysis) that evaluated the use of LIEST for the treatment of erectile dysfunction. One clinical trial evaluated the applicability in Peyronie’s Disease and one other clinical trial evaluated the applicability after radical prostatectomy.

**Conclusions:**

The literature presents little scientific evidence but suggests good results with the use of LIEST for ED. Despite a real optimism since it is a treatment modality capable of acting on the pathophysiology of ED, we must remain cautious, until a larger volume of higher quality studies allows us to establish which patient profile, type of energy and application protocol will achieve clinically satisfactory results.

## INTRODUCTION

In recent years, there has been a substantial increase in the number of low-intensity extracorporeal shock wave therapy (LIEST) studies for erectile dysfunction (ED) (1). This new therapy comes with the hope of being the only modality capable of acting directly on the pathophysiology of ED, offering a remodeling of the erectile tissue and thus some degree of recovery. However, like all new technology, especially those involving very technical aspects such as new devices, different types of energy, with physical aspects that are not familiar to the urologist’s routine, require time and continuous verification to gain the real confidence of the doctors to recommend them. As these are areas that we do not master, we need to be continuously promoting training efforts on the subject and for that reason, we have here, in this 11 article narrative review, the objective of promoting a concise didactic report that brings light to those interested in this new therapeutic modality applied to ED.

**Figure 1:**
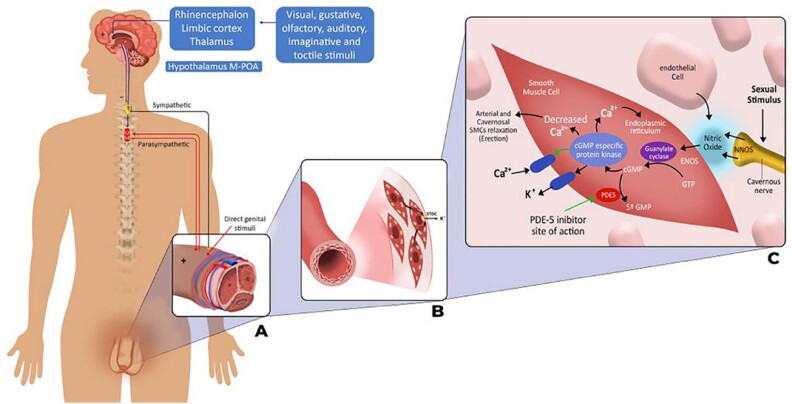
From left to right: The figure demonstrates a coordinated and complex chain of events necessary to obtain an erection, from a stable psychological base, through an intact neuroaxis conducting the stimulus to penile vascular tissue, ultimately reflected in the cellular physiology that leads to the endothelial smooth muscle relaxation process, promoting erection. Any disturbance in one or more chains of this process can lead to erectile dysfunction.

ED is a common condition that affects approximately 18 million adults in the United States (2). It is a condition known to impact not only sex life, but also negatively mental health and overall quality of life (3). The most common type of ED is vasculogenic, which occurs as a result of vascular impairment of penile arteries and/or veins, leading to insufficient blood flow or poor blood retention, usually caused by diseases as high blood pressure, diabetes and dyslipidemia. Didactically, if any of the necessary steps for a normal erection are affected, an erection dysfunction can be triggered. Thus, from psychological factors (anxiety, stress, depression, psychological disorders), to neurogenic impairment, from the neuro-axis at the central level (spinal cord diseases) to peripheral impairment such as it occurs in radical prostatectomy, are causes of ED. We cannot fail to mention endocrine causes as thyroid disease and hypogonadism. This whole set of diseases can occur alone or altogether, ultimately causing, at the cellular level, the penile erectile tissue not to receive the necessary stimulus to initiate the erection process or to become insensitive to it, causing erectile dysfunction in various severity levels (Figure-1).

At the molecular level an erection is initiated when nitric oxide (NO) is released from non-adrenergic and non-cholinergic nerve fibers in response to sexual stimulation. This activates guanylyl cyclase, increasing the concentration of cyclic guanosine monophosphate (cGMP) in the smooth muscle cells of the penis. Simultaneously, parasympathetic cholinergic nerve fibers release acetylcholine, which activates adenylyl cyclase and increases the intracellular concentration of cyclic adenosine monophosphate (cAMP). Calcium levels then decrease, causing smooth muscle cells to relax and increase blood flow. The outflow of blood from the penis is impeded by compression of the subtunic venules, leading to a permanent state of rigidity (Figure-1C).

Another condition worth mentioning is ED as a commonly side effect among patients with prostate cancer who have undergone radical prostatectomy and/or radiotherapy (4-6). The use of nerve-sparing techniques during surgery has been shown to improve recovery of erectile function in many cases, however, some patients still experience ED after undergoing bilateral nerve-sparing radical prostatectomy, which can be caused by mechanical stretching or thermal damage to cavernous nerves, ischemic injury, or local inflammation caused by surgery (7). The increase in fibrosis and the decrease in the elasticity of the erectile tissue in the corpora cavernosa are factors that corroborate the picture. Factors such as age (older), presence of comorbidities, higher prostate-specific antigen (PSA) levels, and worse pretreatment sexual health scores (IIEF score) have been associated with a greater likelihood of developing erectile dysfunction after treatment of prostate cancer. Phosphodiesterase-5 (PDE5i) inhibitors are currently used and have been shown to increase levels of cyclic GMP in penile smooth muscle cells, preserving smooth muscle content and reducing body fibrosis (8, 9). The use of vacuum erection devices, intraurethral suppositories and intracavernous injections do not meet the desired efficacy in penile rehabilitation after radical prostatectomy (10). Thus, what was sought was a method capable of acting on the etiology of erectile dysfunction at the cellular level, leading to local remodeling and thus acting in the various forms of ED (11). We sought to find a method capable of acting on the etiology at the cellular level, leading to local remodeling and thus acting on the various forms of erectile dysfunction.

**Figure 2:**
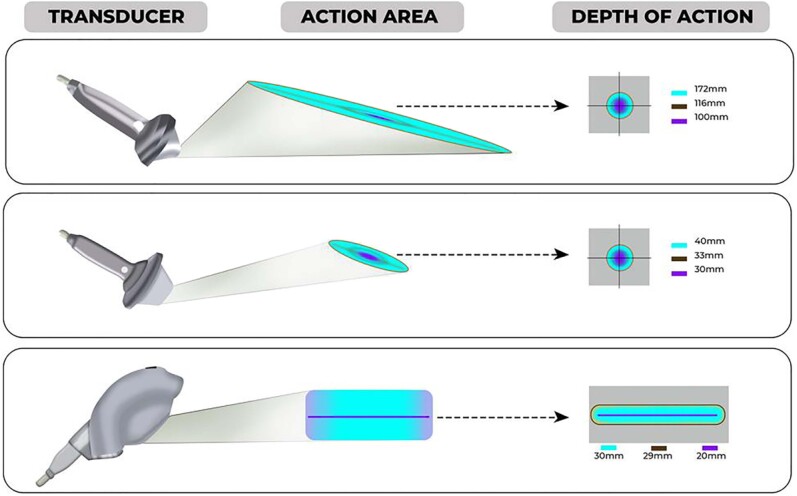
The image reveals the physical concept related between low-intensity shock wave transmission, the shape of the transducer and its tissue interaction. Focal and radial waves can complement each other. While the radial wave is suitable for treating large areas, the focused shock waves can be concentrated deep within the body. In urology, the most modern devices usually emit focal waves through linear transducers, contemplating a larger treatment area in a shorter session time.

**Figure 3:**
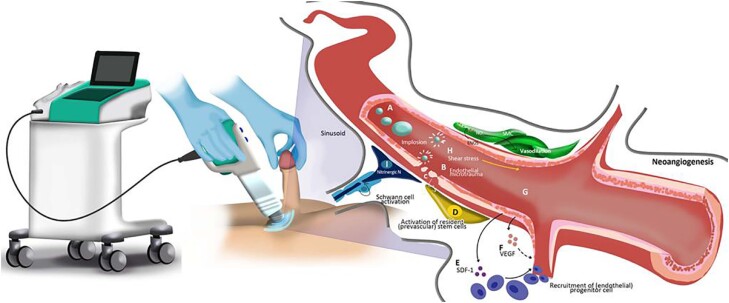
The schematic drawing demonstrates the effect of LIEST at the microscopic level, leading to tissue damage (injury to the vascular endothelium) which, in a second moment, promotes the release and circulation of inflammatory mediators such as - vascular endothelial growth factor (VEGF) - inductors of angiogenesis.

In this scenario came the LIEST, a new treatment option for ED that has shown promising preliminary results (12-14). It differs from other erectile dysfunction treatments, which typically only provide symptom relief, as it works on the underlying pathophysiology of erectile dysfunction. LIEST uses an electro-hydraulic or electromagnetic generator to deliver sound waves directed to the corpora cavernosa and crura, at an energy density of around 0.09 mJ/mm (Figure-2). The therapy was first tested for vasculogenic ED in 2010 by Vardi et al. due to its potential to promote neovascularization in the myocardium (15, 16). LIEST has also been recognized to increase nitric oxide synthesis in penile tissue and support stem cell proliferation (17). Multiple meta-analyses have suggested that LIEST is an effective treatment for erectile dysfunction, resulting in an improvement in the erectile function domain scores of the International Index of Erectile Function (IIEF) and may play a role not only in vasculogenic erectile dysfunction (neoangiogenesis) but also in neurogenesis (18, 19) (Figure-3).

**Figure 4:**
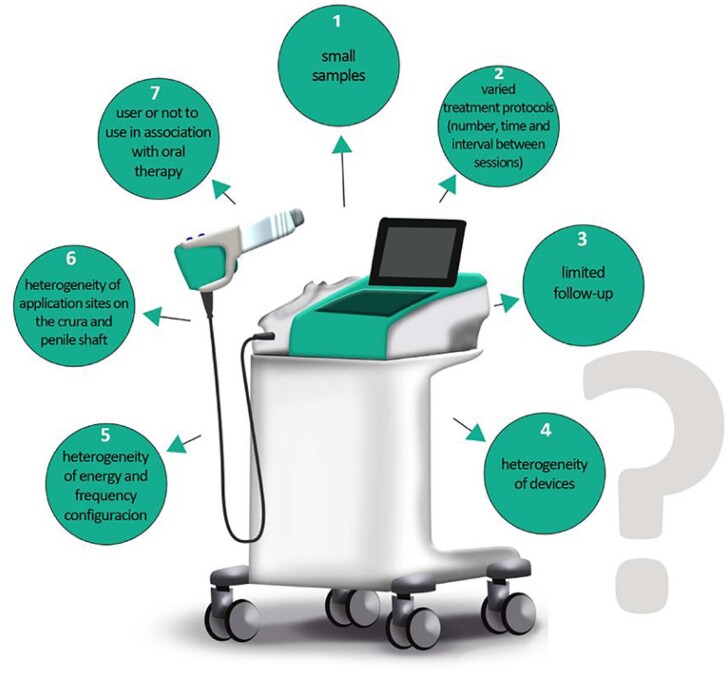
The figure demonstrates the main limitations to LIEST identified in the selected studies during this review. All these aspects altogether prevent, until the present moment, the production of articles of greater scientific relevance.

However, despite the promising results, this literature review suggests that there is a lack of robust data on the use of LIEST for erectile dysfunction, regardless of etiology. This is because the researches have small samples, with varied treatment protocols (number, time and interval between sessions and association or not with oral therapy for ED), limited follow-up, heterogeneity of devices and energy configuration and applied frequency, divergences regarding the application sites, creating an enormous challenge for data interpretation and exclusion of biases making it difficult to draw firm conclusions about the effectiveness of LIEST (Figure-4).

We hope, through this review, to point out which concepts are already consolidated and which paths we still need to advance.

## MATERIALS AND METHODS

We performed a review about the role of shockwave therapy for ED with a bibliographic search on Pubmed restricted to publications from 2010 onwards, using key expressions as “low-intensity extracorporeal shock wave therapy”, “erectile dysfunction” and “randomized controlled trial”. The literature search was carried out independently by VR, SM and FL and consensus on article selection was reached through open discussion. The review included all references to relevant studies and full-text articles in peer-reviewed journals that evaluated the impact of LIEST on erectile dysfunction of any etiology. The present review only included randomized clinical trials, systematic reviews or meta-analyses with a single exception - a cases series - due to its notorious relevance. We included only papers published in English and excluded all case reports, editorials, and opinions of specialists.

## RESULTS

**Figure 5:**
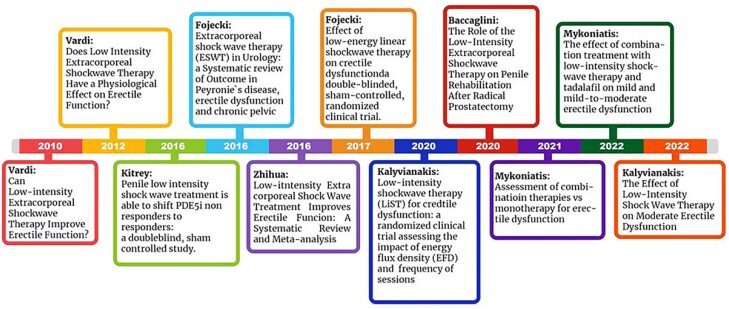
Schematic drawing showing the timeline of the papers of LIEST studied in this review.

In this section we will reveal the findings of the present review. In Figure-5 we can observe the timeline of the selected articles.

## LIEST AND THE ERECTILE FUNCTION - FROM VARDI 2010 TO THE PRESENT: WHAT DO WE KNOW?

Vardi et al. (16) were the first to investigate the use of low-intensity extracorporeal shock wave therapy (LIEST) for the treatment of vasculogenic ED. The aim of this initial study was to evaluate the effectiveness of LIEST in men with erectile dysfunction who had previously responded to oral phosphodiesterase type 5 inhibitors. Twenty men with vasculogenic erectile dysfunction with IIEF - erectile dysfunction domain scores between 5 and 19 and abnormal nocturnal penile tumescence parameters were evaluated. Five distinct sites on the penile shaft and crura were treated with LIEST. Through sexual function questionnaires, nocturnal penile tumescence parameters, penile and systemic endothelial function tests, erectile function was assessed at screening and also one month after the completion of the two therapy sessions. At the 3- and 6-month follow-up visits, the IIEF questionnaire was completed. At the 1-month follow-up, all men had significant increases in IIEF domain scores, which remained constant through 6 months. In addition, significant increases in erection duration and penile rigidity were observed, as well as improvements in penile endothelial function. After a 6-month follow-up, ten men no longer needed the PDE5-I medication. No pain was reported as a result of treatment, and no adverse events were observed during the follow-up period. This was the first study to evaluate the effectiveness of LIEST for ED (16). This method was successful and well tolerated, suggesting a physiological effect on cavernous hemodynamics, a potential to improve erectile function and help with penile rehabilitation without the use of ongoing medications. Although the short-term results were encouraging at the time, we know that there is still a long way to go.

Continuing the investigation, two years after the first publication, Vardi et al. (20) returns with another study, seeking to answer an important question: would LIEST bring any benefit to the group of patients with ED still responsive to oral therapy? To shed light on this topic, he aimed to examine the clinical and physiological effects of LIEST in men with organic erectile dysfunction who were responsive to phosphodiesterase type 5 inhibitors. After a one-month washout period, 67 men were randomly assigned to receive 12 extracorporeal low-intensity shock wave therapy sessions, or sham therapy. Using validated sexual function questionnaires and veno-occlusive tension gauge plethysmography, erectile function and penile hemodynamics were examined before initial therapy and one month after final treatment. In the treated group, the IIEF increased considerably more than in the sham treatment group. Furthermore, 19 men in the treated group who originally could not achieve erections sufficient for penetration were able to do so after therapy, whereas none of the men in the placebo group were able to do so. Penile hemodynamics improved in the treatment group but not in the placebo group. No participants reported experiencing any unwanted effects or discomfort (20). This was the first randomized, double-blind, simulation-controlled study to demonstrate that LIEST has a short-term beneficial clinical and physiological effect on erectile function even in men who respond to oral treatment with phosphodiesterase type 5 inhibitors. At the time, the applicability, tolerability, and potential rehabilitative properties of this treatment made it a viable new alternative therapy for men with ED.

In an interesting study, performed by Kitrey and his group, a simulation-controlled investigation of the effect of low-intensity shock wave penile treatment on patients unable to engage in sexual intercourse using PDE5i (phosphodiesterase type 5 inhibitor) was carried out (21). This prospective, randomized, double-blind, sham-controlled study was conducted in men with vasculogenic erectile dysfunction who discontinued PDE5i treatment due to lack of efficacy (21). With PDE5i, all patients had an erection hardness score of 2 or less. 37 subjects were treated with low-intensity shock waves (12 sessions of 1,500 pulses of 0.09 mJ/mm^2^ at 120 pulses per minute) and 18 patients were treated with a sham probe. 54.1% of patients in the LIEST group and 0% of patients in the placebo group achieved an erection strong enough for vaginal penetration. According to changes in International Index of Erectile Function-Erectile Function (IIEF-EF) scores, treatment was beneficial in 40.5% of men receiving shockwave treatment but in none in the placebo group (p = 0.001). 56.3 percent of patients treated with shockwaves after placebo therapy achieved an erection sufficient for penetration (p< 0.001). As conclusions of this article, they observed that LIEST was beneficial even for patients with severe erectile dysfunction who did not react to PDE5i. As remarkable information in this publication, we should keep that about half of the patients submitted to LIEST were able to achieve an erection strong enough for penetration after treatment with PDE5i (21).

**Table 1:** Data summary.

Author	Design	N	Device	Energy	Protocol	Session
**Vardi et al., 2010** (16)	Case Series	20	Omnispec ED1000 electrohydraulic device - Medispec	0.09 mJ/mm^2^	– 300 pulses per treatment point. – frequency of 120p/min.	>>> 2x/wk for 3 weeks >>> 3 week interval >>> 2x/wk for 3 weeks
**Vardy et al., 2012** (20)	Randomized, double-Blind, sham controlled study	60	Omnispec ED1000 electrohydraulic device - Medispec	0.09 mJ/mm^2^	– 300 pulses per treatment point – frequency of 120p/min.OrSham group	>>> 2x/wk for 3 weeks >>> 3 week interval >>> 2x/wk for 3 weeks
**Kitrey et al., 2016** (21	Randomized, double-blind, sham controlled study	53	Omnispec ED1000 electrohydraulic device - Medispec	0.09 mJ/mm^2^	– 300 pulses per each 5 treatment point – frequency of 120p/min.Or Sham group	>>> 2x/wk for 3 weeks >>> 3 week interval >>> 2x/wk for 3 weeks
**Fojecki et al., 2017** (22)	Systematic review	238	Depending on the selected study	Depending on the selected study	Depending on the selected study	Varying according to the selected study
**Zhihua et al., 2016** (23)	Systematic review /Meta-analysis	833	Depending on the selected study	Depending on the selected study	Depending on the selected study	Varying according to the selected study
**Fojecki et al., 2017** (24)	Randomized, double-blinded, sham-controlled study	118	FBL10, Richard-Wolf GmbH,	0.09 mJ/mm^2^	– 300 pulses per treatment point at penile shaft – 150 pulses per treatment point at each crura – frequency of 300p/minOrSham group	>>> 1x/wk for 5 weeks >>> 4 week interval >>> 1x/wk for 5 weeks
**Kalyvianakis et al., 2019** (25)	Randomized, four parallel arms, open-label study	89	ARIES 2 Smart focus probe – Dornier	0.05mJ/mm^2^ (Groups A and B) 0.10 mJ/mm^2^ (C and D)	– 1000 pulses per penile treatment point and crura plus; – 500 pulses to the left and right proximal penile shaft.Frequency 640p/min (Groups A and B)Frequency 300 p/min (Groups C and D)	>>> 2 or 3x/wk for 12 weeks >>> no interval
**Baccaglini, 2020**	Randomized, two parallel arms, open-label study	**77**	Renova	0.09 mJ/mm^2^	– 600 pulses per treatment point at penile shaft plus – 150 pulses per treatment point at each crura – frequency of 300p/min.	>>> 1x/wk for 8 weeks after radical prostatectomy
**Mykoniatis, 2021**	Systematic review /Meta-analysis	3853	Depending on the selected study	Depending on the selected study	Depending on the selected study	Varying according to the selected study
**Mykoniatis, 2022**	Double-blind, randomized, placebo -controlled clinical trial	50	–	0.09 mJ/mm^2^	–	>>> 2x/wk for 3 weeks >>> no interval
**Kalyvianakis, 2022**	Double-blind, randomized, sham-controlled clinical trial	67	ARIES 2 Smart focus probe - Dornier	0.09 mJ/mm^2^	– Total of 5000 pulses delivered at the corpora cavernosa, to the crura cavernosa and to the penile hila. – Frequency 300 p/min	>>> 2x/wk for 6 weeks

Expanding the range of possibilities for using shockwave therapy Fojecki conducted an important study analyzing the possible use of LIEST in Peyronie’s Disease and Chronic Pelvic Pain (22). This research aimed to analyze high quality evidence studies on the use of LIEST for urological diseases. The bibliographic search included EMBASE, Medline and PubMed databases, looking for randomized and controlled studies. The systematic review was performed according to PRISMA principles. At the time, 10 studies were identified for three urological indications: erectile dysfunction, Peyronie’s disease and chronic pelvic pain. Four ED studies including 337 participants were considered and, according to IIEF-EF and EHS data, LIEST had a substantial favorable effect on PDE-5i responders. Two of the three Peyronie’s disease (PD) studies comprising 238 patients revealed a reduction in pain, although neither penile deviation nor plaque size changed significantly. Three studies involving 200 men and persistent pelvic pain (CPP) found improvements on the National Institutes of Health Chronic Prostatitis Symptom Index (NIH-CPSI). In terms of treatment approaches and outcome measures, there was substantial heterogeneity between studies, making it difficult to compare results, but the authors should be congratulated for their broad view and foresight in applicability of LIEST (22).

Returning to our focus, previous research has shown LIEST to be an effective treatment for ED. But did all studies in the literature corroborate the prior evidence and effectiveness of LIEST? And what would be clinical efficacy? In the lines below we will add some interesting information to improve your clinical judgment.

Zhihua Lu et al, together with collaborators including Tom F. Lue, provided an overview of the literature in 2016, through a meta-analysis that consolidated some relevant aspects of the therapy through a scientific compilation of studies that suggested that LIEST could significantly improve the IIEF and EHS of patients with erectile dysfunction. To this end, a complete search in the PubMed and Embase databases was carried out with data up to November 2015 (23). Patients with erectile dysfunction were included in the studies and the IIEF and the EHS were the most used instruments to assess therapeutic efficacy (23). From 2005 to 2015, 14 surveys including 833 individuals undergoing therapy were compiled and synthesized. Seven surveys were randomized clinical trials (RCTs), with the important bias that in these publications the types of machines, configurations, and application protocols varied among themselves. Regardless of the foregoing, the meta-analysis demonstrated that LIEST can significantly increase IIEF and EHS with at least three months of proven therapeutic efficacy, with therapeutic efficacy for individuals with mild to moderate ED being superior to that of patients with severe ED or the presence of comorbidities. Another striking point of the study was that the energy flux density, the number of shock waves per session and the duration of the LIEST treatment were directly related to the clinical result, i.e., the greater the energy applied, the better the effect seemed to be. Regardless of differences in LI-ESWT configuration factors or treatment procedures, most of these investigations demonstrated positive results, and thus, these findings would indicate that LIEST would have the potential to considerably increase the IIEF and EHS of patients with ED, bringing greater confidence in the use of the method. Naturally, the authors point out that the release of robust evidence from more RCTs and longer-term follow-up would further increase confidence in the use of LIEST and that, therefore, it should go down the path of drawing more robust conclusions.

Advancing in knowledge, in 2017 Fojecki returned with this paper that raised questions about the effectiveness of LIEST (24). The aim here, once again, was to investigate the effectiveness of LIEST in the treatment of erectile dysfunction, and for this purpose, the study included 126 men with erectile dysfunction with an IIEF score below 25. These participants were separated into two groups, with one group receiving LIEST once a week for five weeks and the other receiving a placebo. After a four-week hiatus, both groups undergo five weeks of active treatment once a week. At baseline, after 9 weeks, and after 18 weeks, participants completed the IIEF, EHS, Sexual Quality of Life-Men, and the Satisfaction Inventory for Erectile Dysfunction. The primary outcome was an increase of at least five points in the IIEF-EF score (clinical efficacy), and the secondary outcome was an increase in the EHS score. In the sham group, the mean IIEF-EF score increased from 11.5 at baseline to 13.0 after five sessions and 12.6 after 10 sessions, while in the LIEST group, the mean IIEF-EF score increased from 10.9 at the beginning to 13.1 after five sessions and 11.8 after 10 sessions (24). This study concluded that LIEST has no clinically significant effect on erectile dysfunction.

In 2020, Kalyvianakis presented a concept that urologists have little mastered: energy flux density (EFD) (25). This is a fundamental concept which, until now, has not been studied in relation to LIEST for erectile dysfunction and that must be understood by urologists. The amount of energy distributed according to the focal area must be known to allow a treatment schedule that contemplates the entire corpora cavernosa and crura with the same amount of energy and not occasionally sparsely, with energies distributed in a dispersed way, and with a total load of energy per point varying from point to point. In this interesting study, he examined the efficacy and safety of 12 treatment sessions at EFD 0.05 versus 0.10 mJ/mm^2^ when applied twice or three times a week (25). Patients using PDE5 inhibitors with vasculogenic erectile dysfunction were randomized into four groups to receive 12 sessions of LIEST. Group A (n = 24) received two sessions per week with an EFD of 0.05 mJ/mm^2^; Group B (n = 24) received three sessions per week with EFD of 0.05 mJ/mm^2^; Group C (n = 24) received two sessions per week with EFD of 0.10 mJ/mm^2^; and Group D (n = 25) received three sessions per week with an EFD of 0.10 mJ/mm^2^. IIEF-EF, clinical findings, and triplex doppler ultrasonography findings were employed to assess erectile function. A total of 89 patients completed the 6-month follow-up assessment, and in all four groups, improvement was identified in terms of the mean IIEF-EF score. It is worth noting that there were no statistically significant differences between the frequency of sessions, but a trend towards greater efficacy was observed with EFD 0.10 mJ/mm^2^ (a greater quantity), although without statistical significance. It is critical here that the study was carried out without a control group, but bringing an interesting aspect that patients can equally benefit from two or three therapy sessions per week and, if provided with a higher energy flow (EFD of 0.10 mJ/mm^2^), in theory, would produce superior effects.

After this initial compilation of the literature, we have reached an exciting point in our analysis. Considering the recently demonstrated benefits of applying LIEST in vasculogenic ED, expectations arise regarding its possible applicability in penile rehabilitation. Would there be space? Would it make sense even when dealing with a dysfunction with a neurogenic component? Given the role of LIEST in angiogenesis and neurogenesis, it is believed that this therapy may be of benefit in the treatment of prostatectomy-induced ED (26). The objective of the study of Baccaglini et al. (27) was precisely to evaluate the response to the early introduction of phosphodiesterase-5 inhibitors in association with LIEST in patients after radical prostatectomy. In this robust randomized clinical trial, the authors performed two parallel arms with an allocation ratio of 1:1. Immediately after removal of the transurethral catheter, both groups received tadalafil at a dose of 5 mg/day, and the experimental group received 2,400 shocks/session-week distributed over four penile areas (27). The primary clinical endpoint was a 4-point difference in favor of the experimental group based on IIEF-5. 92 men were enrolled in the study between September 25, 2017, and December 3, 2018, and when comparing the final mean IIEF-5 scores of the two groups, a significant difference was observed (P = 0.006). However, the main clinical endpoint requiring a 4-point difference between the arms has not yet been reached, and therefore, in terms of clinical benefit, the use of LIEST for penile rehabilitation remains uncertain.

As we know, an ocean of ED therapies has emerged in recent years. But would they together offer a synergy capable of improving overall efficiency? Surfing a wave in search of evaluating whether monotherapy versus combinations of therapies would present better results, we found in an important paper by Mykonatis and his group (28). The study aimed to determine whether different combined treatments for ED are associated with better outcomes than first-line monotherapy for erectile dysfunction in subgroups of individuals with erectile dysfunction. To this end, a search of randomized clinical trials in MEDLINE, the Cochrane Library, and Scopus was carried out, considering studies up to October 10, 2020, and taking into account the PRISMA (Preferred Reporting Items for Systematic Reviews and Meta-analysis) criteria. Separate analyses were conducted for the mean change in the IIEF score from baseline and the number of adverse events by treatment modality and patient subgroup. The authors identified 44 studies involving 3,853 men with a mean age of 55.8 years. As conclusions, we emphasize that this study revealed that the combination of PDE5 inhibitors and antioxidants improved erectile dysfunction without increasing adverse effects (28). PDE5 inhibitor treatment with daily tadalafil, shock waves, or a vacuum device was associated with even greater improvement; however, it was difficult to extract the participation of each of the treatments in the overall efficacy. Still, the data presented indicate that combination therapy is safe, associated with better outcomes, and should be considered a first-line treatment, especially for refractory, complex, or difficult-to-treat ED.

Coming to the end, during our selection of publications, we identified another important paper, published in 2022 in the J*ournal of Sexual Medicine* that we believe is worth mentioning (29). In it, Mykoniatis conducted a double-blind, randomized, placebo-controlled clinical trial involving 50 patients to determine the efficacy and safety of combination therapy using LIEST and tadalafil in the treatment of mild to moderate vasculogenic erectile dysfunction. Patients were divided into two groups, with one group receiving LIEST twice a week for three weeks and tadalafil once daily for four weeks, and the other group receiving LIEST and a placebo. The primary outcome measure was the change in the IIEF-EF domain score from baseline three months after treatment. Erectile function was also assessed at one and six months. The number of patients who achieved a minimal clinically important difference in IIEF-EF, as well as the safety of the combination therapy, were also evaluated, and the results showed that combination therapy with LIEST and tadalafil led to a statistically significant improvement in IIEF-EF scores at three and six months compared to LIEST monotherapy (29). The number of patients achieving a minimal clinically important difference in the IIEF-EF also significantly increased in the combination therapy group at three months. No adverse events were reported during the study, and as described, the findings strongly suggest that LIEST and tadalafil combination therapy may be more effective in improving erectile function in patients with mild or mild to moderate vasculogenic ED than LIEST monotherapy. Certainly, more research on this topic must be carried out to confirm these results, but it seems that an important question begins to be answered here.

Closing our sequence of articles, in this last, unprecedented, and recent study produced by Dimitrios Kalyvianakis and his group, we were presented with the first double-blind, randomized, and placebo-controlled trial to evaluate the efficacy and safety of LIEST in patients with moderate ED (30). Seventy patients in the presence of moderate vasculogenic ED underwent a 1-month PDE5 inhibitor washout period, documented with a score between 11 e 16 on the IIEF. These selected patients were randomized to receive 12 sessions of LIEST (35 patients) or sham therapy (35 patients) twice a week (30). At the end, the authors demonstrated that in patients with moderate erectile dysfunction, twelve sessions of LIEST twice a week for six weeks using a treatment plan of 5000 pulses with an energy flux density of 0.09 mJ/mm^2^ and frequency of 5 Hz are highly recommended. The machine used was an ARIES 2TM probe with Smart Focus. In this important clinical trial, it was observed that more than two-thirds of patients who performed LIEST sessions twice a week for 6 weeks had a clinically significant difference in IIEF. With this, we compose one more evidence that a standardized treatment can produce effective and promising results in the short term.

## CONCLUSIONS

We are taking our initial steps towards an adjunctive treatment for ED that appears to be effective and safe. Despite the great enthusiasm with the new therapy, the present literature indicates that we are not yet facing the discovery of gunpowder in terms of the treatment of erectile dysfunction. We must remain optimistic but cautious until a larger volume of studies of higher quality allow us to establish which patient profile, type of energy, and ideal application protocol will achieve clinically satisfactory results. There will certainly be clinical space for LIEST, but as we are dealing with a new technology with fleeting and short-term results, we must clarify in detail these related aspects to our patients, making a transparent and shared decision. LIEST seems to be here to stay, but there is still a long way to go.
